# Supplementary material for: SARS-CoV-2 N protein-induced Dicer, XPO5, SRSF3, and hnRNPA3 downregulation causes pneumonia
Source: Nat Commun. 2024 Aug 13;15:6964. doi: 10.1038/s41467-024-51192-1 (PMC11322655; doi:10.1038/s41467-024-51192-1)
Supplement: Supplementary file 1 — Supplementary_information [file 41467_2024_51192_MOESM1_ESM.pdf]

# Supplementary Information

**SARS-CoV-2 N protein-induced Dicer, XPO5, SRSF3, and hnRNPA3 downregulation causes pneumonia**

Yu-Wei Luo[1#], Jiang-Peng Zhou[1#], Hongyu Ji[1#], Doudou Xu[2#], Anqi Zheng[3], Xin Wang[1], Zhizheng Dai[1], Zhicheng Luo[1,3], Fang Cao[1], Xing-Yue Wang[3], Yunfang Bai[1], Di Chen[1], Yueming Chen[3], Qi Wang[4], Yaying Yang[5], Xinghai Zhang[6], Sandra Chiu[7,8], Xiaozhong Peng[2,9]*, Ai-Long Huang[1]*, Kai-Fu Tang[1]*

[1] Key Laboratory of Molecular Biology on Infectious Disease, Ministry of Education, Chongqing Medical University, Chongqing, P.R. China

[2] State Key Laboratory of Respiratory Health and Multimorbidity, Key Laboratory of Pathogen Infection Prevention and Control (Peking Union Medical College), Ministry of Education, National Center of Technology Innovation for animal model, Institute of Laboratory Animal Sciences, CAMS & PUMC, Beijing, P.R. China

[3] Key Laboratory of Diagnosis and Treatment of Severe Hepato-Pancreatic Diseases of Zhejiang Province, The First Affiliated Hospital of Wenzhou Medical University, Wenzhou, Zhejiang, P.R. China

[4] Department of Basic Medicine, Chongqing Medical University, Chongqing, P.R. China

[5] Department of Pathology, Molecular Medicine and Cancer Research Center, Molecular Medicine Diagnostic and Testing Center, Chongqing Medical University, Chongqing, P.R. China

[6] State Key Laboratory of Virology, Wuhan Institute of Virology, Center for Biosafety Mega-Science, Chinese Academy of Sciences, Wuhan, Hubei, P.R. China

[7] Division of Life Sciences and Medicine, University of Science and Technology of China, Hefei,

Anhui, P.R. China

[8] Key Laboratory of Anhui Province for Emerging and Reemerging Infectious Diseases, Hefei, Anhui, P.R. China

[9] Institute of Basic Medical Sciences, Chinese Academy of Medical Sciences, School of Basic Medicine Peking Union Medical College, Beijing, P.R. China

[#] These authors contributed equally.

***Corresponding authors:**

Email: tangkaifu@cqmu.edu.cn; ahuang@cqmu.edu.cn; pengxiaozhong@pumc.edu.cn

**This PDF file includes:**

Supplementary Figures 1 to 10

Supplementary Tables 1 to 4

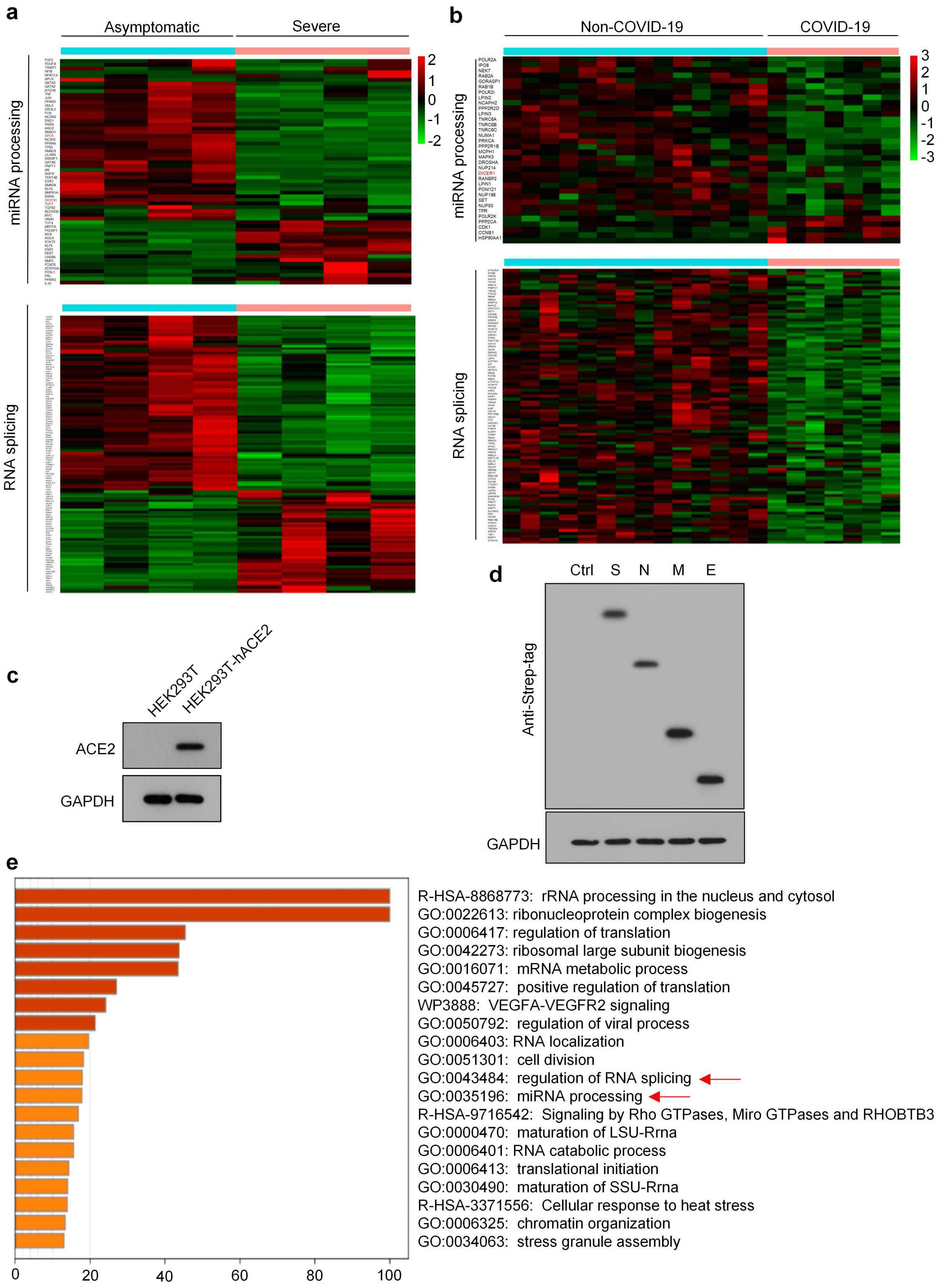
a
Asymptomatic
Severe
miRNA processing
RNA splicing
2
1
0
-1
-2
b
Non-COVID-19
COVID-19
miRNA processing
RNA splicing
3
2
1
0
-1
-2
-3
c
HEK293T
HEK293T-hACE2
ACE2
GAPDH
d
Ctrl
S
N
M
E
Anti-Strep-tag
GAPDH
e
R-HSA-8868773: rRNA processing in the nucleus and cytosol
GO:0022613: ribonucleoprotein complex biogenesis
GO:0006417: regulation of translation
GO:0042273: ribosomal large subunit biogenesis
GO:0016071: mRNA metabolic process
GO:0045727: positive regulation of translation
WP3888: VEGFA-VEGFR2 signaling
GO:0050792: regulation of viral process
GO:0006403: RNA localization
GO:0051301: cell division
GO:0043484: regulation of RNA splicing
GO:0035196: miRNA processing
R-HSA-9716542: Signaling by Rho GTPases, Miro GTPases and RHOBTB3
GO:0000470: maturation of LSU-Rrna
GO:0006401: RNA catabolic process
GO:0006413: translational initiation
GO:0030490: maturation of SSU-Rrna
R-HSA-3371556: Cellular response to heat stress
GO:0006325: chromatin organization
GO:0034063: stress granule assembly
0
20
40
60
80
100

**Supplementary Figure 1. RNAi components and splicing factors are downregulated in patients with severe COVID-19 and may interact with SARS-CoV-2 N protein;** related to Fig. 1. **(a)** Heatmap depicting the mRNA levels of RNAi components and RNA splicing factors in monocytic-myeloid-derived suppressor cells (M-MDSCs) from patients with severe or asymptomatic coronavirus disease 2019 (COVID-19). RNA sequencing data were downloaded from the Gene Expression Omnibus (GEO) database (GSE178824). **(b)** Heatmap depicting the mRNA levels of RNAi components and RNA splicing factors in lung tissues from deceased patients with COVID-19 and individuals without COVID-19. RNA sequencing data were downloaded from the GEO database (GSE159585). **(c)** Ectopic expression of hACE2 in HEK293T cells was confirmed via immunoblotting (n = 3 biological replicates). **(d)** HEK293T cells co-transfected with plasmids expressing different strep-tagged viral proteins. Immunoblotting with the anti-strep-tag antibody was performed 48 h post-transfection (n = 3 biological replicates). **(e)** Metascape pathway enrichment of putative N protein interactors. Ctrl: control plasmid; N: N protein; S: spike protein; M: membrane protein; E: envelope protein. Source data are provided in the Source Data file.

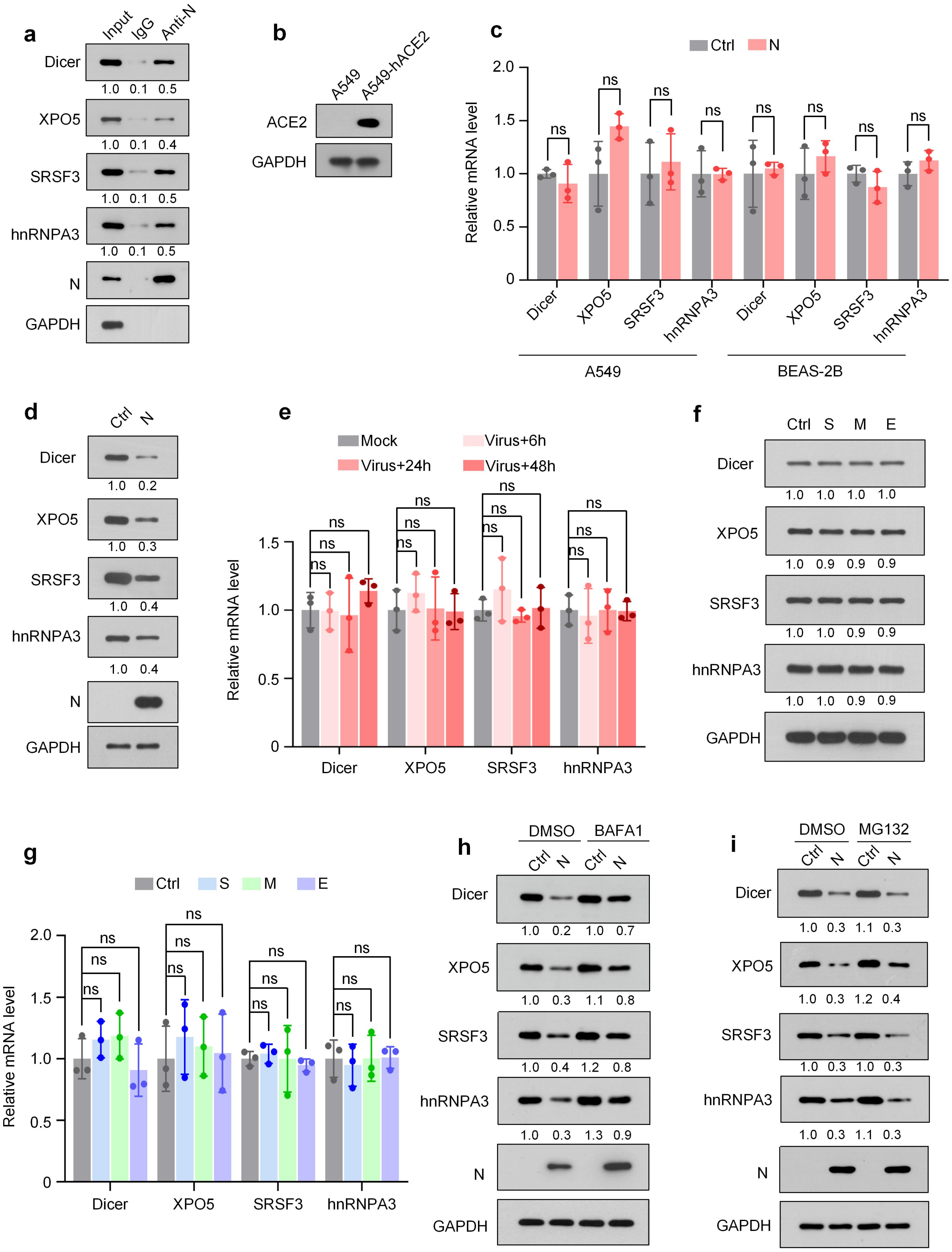


**Supplementary Figure 2. SARS-CoV-2 N protein interacts with Dicer, XPO5, SRSF3, and**

**hnRNPA3 and induces their autophagic degradation;** related to Fig. 1. **(a)** Lysates of BEAS-2B cells stably expressing N protein (BEAS-2B-N) were immunoprecipitated using an anti-N protein antibody and immunoblotted with the indicated antibodies. **(b)** Ectopic expression of hACE2 in A549 cells was confirmed via immunoblotting. **(c)** mRNA levels of *Dicer, XPO5, SRSF3*, and *hnRNPA3* in A549 cells stably transfected with a control plasmid (A549-Ctrl), A549 cells stably expressing N protein (A549-N), and BEAS-2B cells stably transfected with a control plasmid (BEAS-2B-Ctrl) and BEAS-2B-N cells. **(d)** Abundance of Dicer, XPO5, SRSF3, and hnRNPA3 proteins in BEAS-2B-Ctrl and BEAS-2B-N cells. **(e)** A549-hACE2 cells were infected with SARS-CoV-2 or mock; the mRNA levels of Dicer, XPO5, SRSF3, and hnRNPA3 were determined at indicated time points after infection. **(f, g)** A549 cells transfected with plasmids expressing spike (S), membrane (M), and envelope (E) proteins were subjected to immunoblotting (f) and reverse transcription-quantitative polymerase chain reaction (RT-qPCR) (g) 48 h post-transfection. **(h)** Abundance of Dicer, XPO5, SRSF3, and hnRNPA3 proteins in A549-Ctrl or A549-N cells treated with or without bafilomycin A1 (BAFA1). **(i)** Immunoblotting of the indicated proteins in A549-Ctrl or A549-N cells treated with or without a proteasome inhibitor (MG132). The numbers below the blots indicate the relative densitometric quantification of the bands normalized to input (a) or GAPDH bands (d, f, h, i); the mean values in three independent experiments are shown. Data in (c, e, g) are expressed as mean ± SD of three biological replicates. ns, not significant ($p > 0.05$; two-tailed unpaired Student's *t*-test). Ctrl: control plasmid; N: N protein; anti-N: anti-N protein antibody. Source data and exact *p* values are provided in the Source Data file.

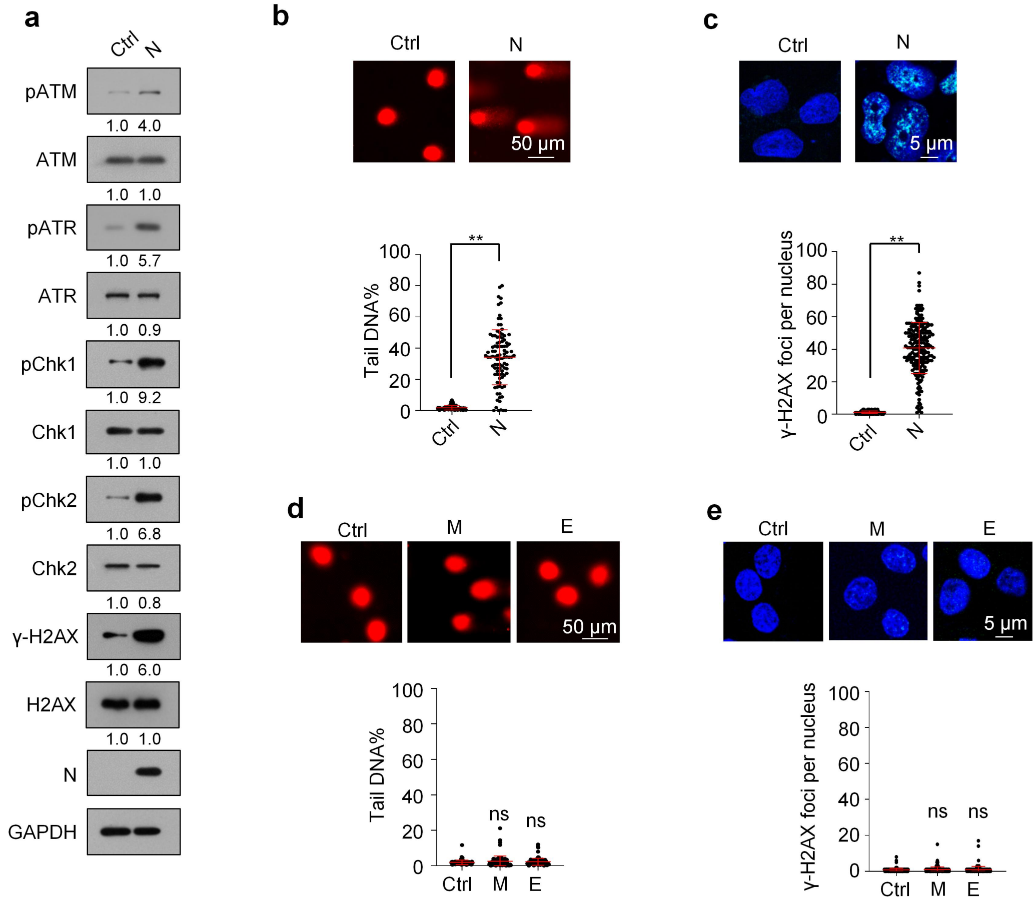


**Supplementary Figure 3. SARS-CoV-2 N protein induces DNA damage;** related to Fig. 3. **(a–c)** DNA damage in BEAS-2B-Ctrl or BEAS-2B-N cells was determined via immunoblotting analysis of the phosphorylation levels of ATM, ATR, Chk1, Chk2, and H2AX (a), comet assay (b), and immunofluorescence with anti-γ-H2AX antibody (c). **(d, e)** A549 cells transfected with plasmids expressing membrane (M) and envelope (E) proteins were subjected to comet assay (d) and immunofluorescence with anti-γ-H2AX antibody (e). Numbers below the blots in (a) represent the relative densitometric quantification of bands normalized to the GAPDH bands; the mean values of bands in three independent experiments are shown. Data in (b–e) are expressed as mean ± SD. n = 100 (b, d) or 200 (c, e) cells from three biological replicates. $^{**}p < 0.01$; ns, not significant ($p > 0.05$; two-tailed unpaired Student's *t*-test). Ctrl: control plasmid; N: N protein.

Source data and exact p values are provided in the Source Data file.

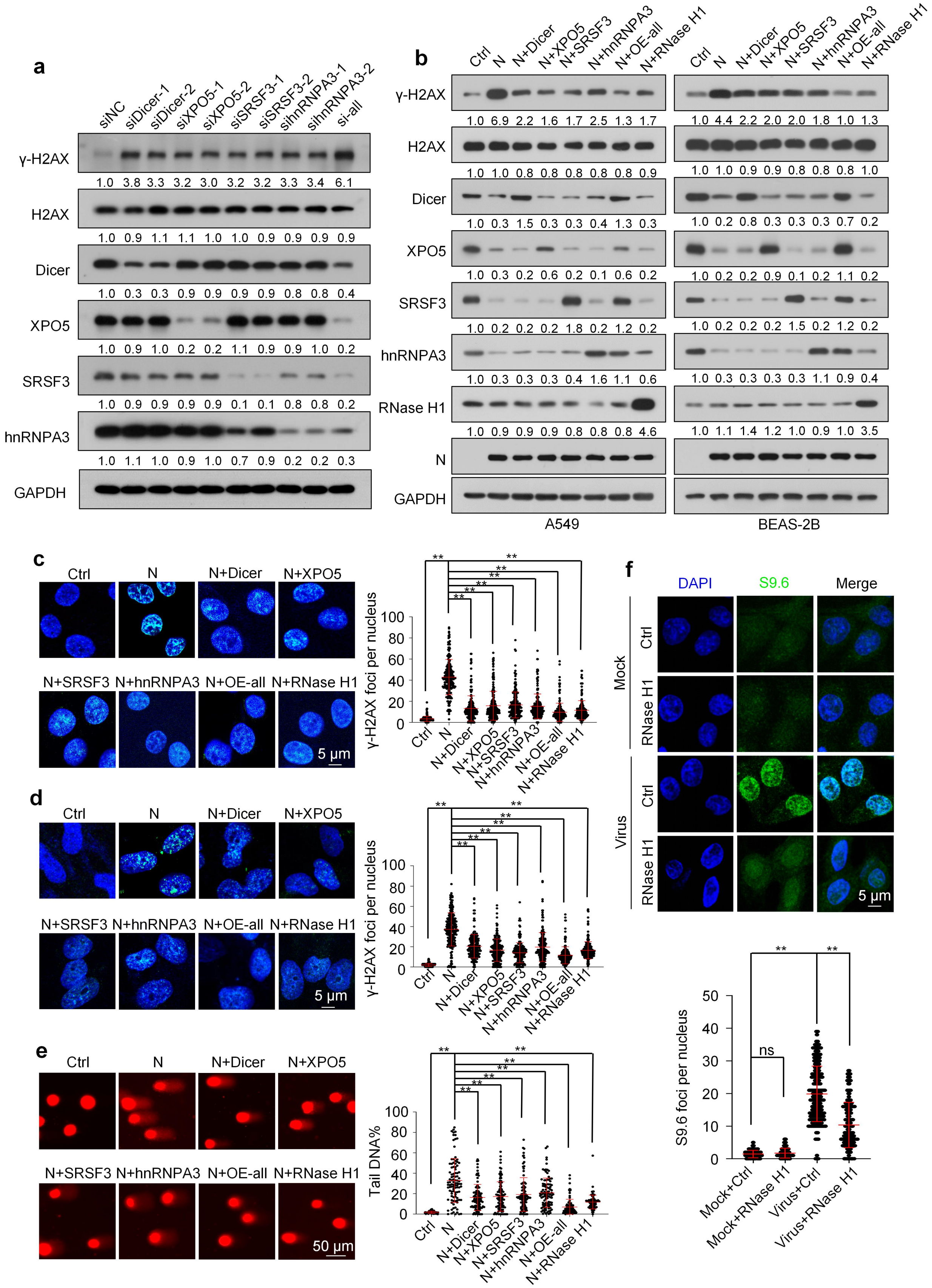
a
siNC
siDicer-1
siDicer-2
siXPO5-1
siXPO5-2
siSRSF3-1
siSRSF3-2
sihnRNPA3-1
sihnRNPA3-2
si-all
γ-H2AX
1.0 3.8 3.3 3.2 3.0 3.2 3.2 3.3 3.4 6.1
H2AX
1.0 0.9 1.1 1.1 1.0 1.0 0.9 0.9 0.9 0.9
Dicer
1.0 0.3 0.3 0.9 0.9 0.9 0.9 0.8 0.8 0.4
XPO5
1.0 0.9 1.0 0.2 0.2 1.1 0.9 0.9 1.0 0.2
SRSF3
1.0 0.9 0.9 0.9 0.9 0.1 0.1 0.8 0.8 0.2
hnRNPA3
1.0 1.1 1.0 0.9 1.0 0.7 0.9 0.2 0.2 0.3
GAPDH
b
Ctrl
N
N+Dicer
N+XPO5
N+SRSF3
N+hnRNPA3
N+OE-all
N+RNase H1
γ-H2AX
1.0 6.9 2.2 1.6 1.7 2.5 1.3 1.7
1.0 4.4 2.2 2.0 2.0 1.8 1.0 1.3
H2AX
1.0 1.0 0.8 0.8 0.8 0.8 0.8 0.9
1.0 1.0 0.9 0.9 0.8 0.8 0.8 1.0
Dicer
1.0 0.3 1.5 0.3 0.3 0.4 1.3 0.3
1.0 0.2 0.8 0.3 0.3 0.3 0.7 0.2
XPO5
1.0 0.3 0.2 0.6 0.2 0.1 0.6 0.2
1.0 0.2 0.2 0.9 0.1 0.2 1.1 0.2
SRSF3
1.0 0.2 0.2 0.2 1.8 0.2 1.2 0.2
1.0 0.2 0.2 0.2 1.5 0.2 1.2 0.2
hnRNPA3
1.0 0.3 0.3 0.3 0.4 1.6 1.1 0.6
1.0 0.3 0.3 0.3 0.3 1.1 0.9 0.4
RNase H1
1.0 0.9 0.9 0.9 0.8 0.8 0.8 4.6
1.0 1.1 1.4 1.2 1.0 0.9 1.0 3.5
N
GAPDH
A549
BEAS-2B
c
Ctrl
N
N+Dicer
N+XPO5
N+SRSF3
N+hnRNPA3
N+OE-all
N+RNase H1
5 μm
γ-H2AX foci per nucleus
**
d
Ctrl
N
N+Dicer
N+XPO5
N+SRSF3
N+hnRNPA3
N+OE-all
N+RNase H1
5 μm
γ-H2AX foci per nucleus
e
Ctrl
N
N+Dicer
N+XPO5
N+SRSF3
N+hnRNPA3
N+OE-all
N+RNase H1
50 μm
Tail DNA%
f
DAPI
S9.6
Merge
Mock
Virus
Ctrl
RNase H1
5 μm
S9.6 foci per nucleus
ns
Mock+Ctrl
Mock+RNase H1
Virus+Ctrl
Virus+RNase H1

**Supplementary Figure 4. SARS-CoV-2 N protein induces DNA damage by downregulating Dicer, XPO5, SRSF3, and hnRNPA3;** related to Fig. 3. **(a)** A549 cells were transfected with the indicated siRNAs and subjected to immunoblotting. **(b)** A549-Ctrl and A549-N cells (left) or BEAS-2B-Ctrl and BEAS-2B-N cells (right) transfected with a control plasmid or plasmids overexpressing Dicer, XPO5, SRSF3, hnRNPA3, or RNase H1 were subjected to immunoblotting. **(c)** A549-Ctrl and A549-N cells transfected with a control plasmid or plasmids expressing Dicer, XPO5, SRSF3, hnRNPA3, or RNase H1 were subjected to immunofluorescence with anti-γ-H2AX antibody. **(d, e)** BEAS-2B-Ctrl and BEAS-2B-N cells transfected with a control plasmid or plasmids expressing Dicer, XPO5, SRSF3, hnRNPA3, or RNase H1 were subjected to immunofluorescence with anti-γ-H2AX antibody (d) and comet assay (e). **(f)** A549-hACE2 cells were transfected with a plasmid expressing RNase H1 or control plasmid and infected with SARS-CoV-2 or mock, and R-loop was detected using immunofluorescence with the S9.6 anti-DNA-RNA hybrid antibody. The numbers below the blots indicate the relative densitometric quantification of the bands normalized to GAPDH bands (a, b); the mean values in three independent experiments are shown. Data in (c–f) are expressed as mean ± SD. n = 100 (e) or 200 (c, d, f) cells from three biological replicates. $^{**}p < 0.01$; ns, not significant ($p > 0.05$; two-tailed unpaired Student's *t*-test). Ctrl: control plasmid; N: N protein; OE-all: cells transfected with Dicer-, XPO5-, SRSF3-, and hnRNPA3-expressing plasmids together. Source data and exact *p* values are provided in the Source Data file.

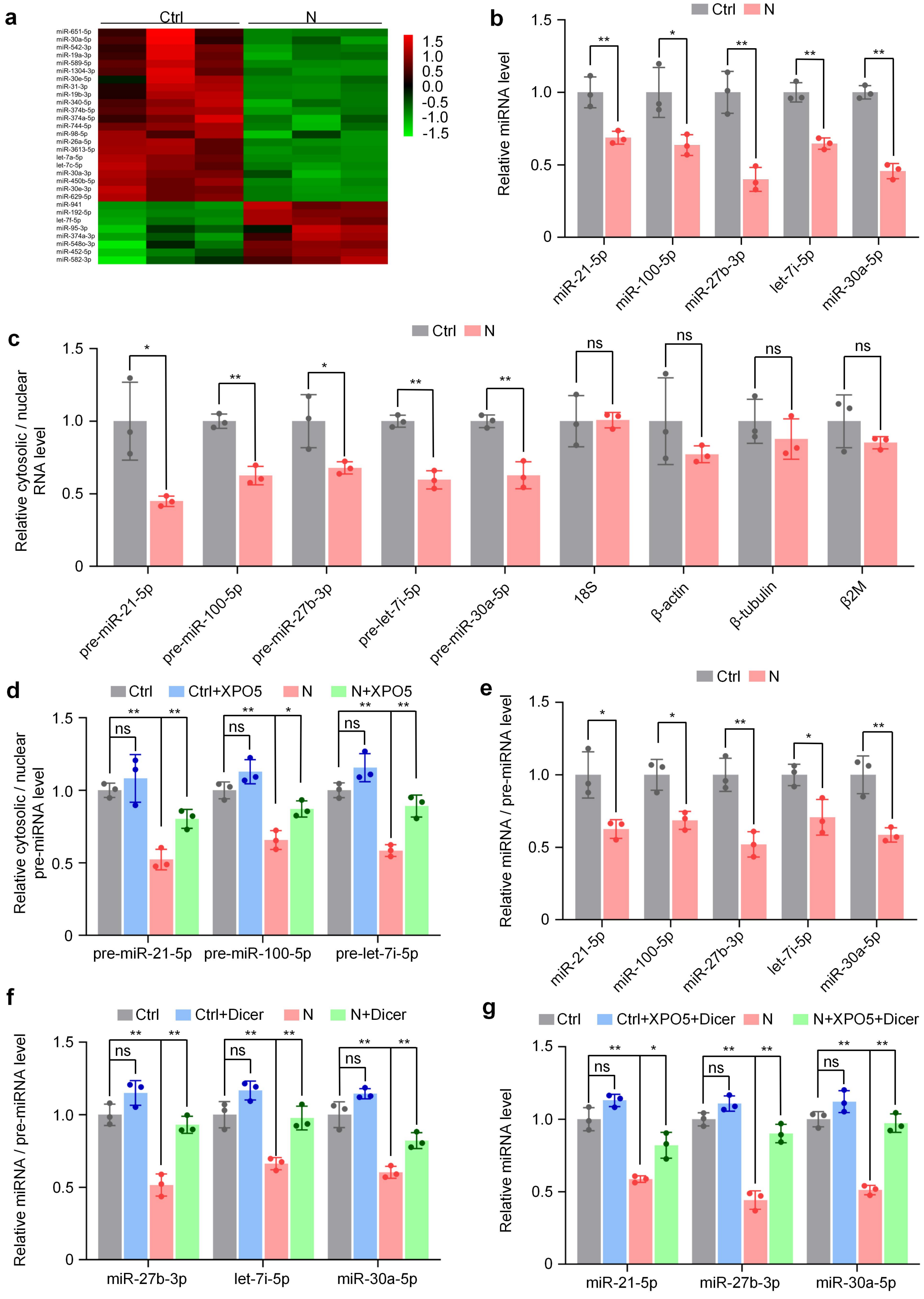

a
Ctrl
N
miR-651-5p
miR-30a-5p
miR-542-3p
miR-19a-3p
miR-589-5p
miR-1304-3p
miR-30e-5p
miR-31-3p
miR-19b-3p
miR-340-5p
miR-374b-5p
miR-374a-5p
miR-744-5p
miR-98-5p
miR-26a-5p
miR-3613-5p
let-7a-5p
let-7c-5p
miR-30a-3p
miR-450b-5p
miR-30e-3p
miR-629-5p
miR-941
miR-192-5p
let-7f-5p
miR-95-3p
miR-374a-3p
miR-548o-3p
miR-452-5p
miR-582-3p
1.5
1.0
0.5
0.0
-0.5
-1.0
-1.5
b
Ctrl
N
Relative miRNA level
miR-21-5p
miR-100-5p
miR-27b-3p
let-7i-5p
miR-30a-5p
c
Relative cytosolic / nuclear RNA level
pre-miR-21-5p
pre-miR-100-5p
pre-miR-27b-3p
pre-let-7i-5p
pre-miR-30a-5p
18S
β-actin
β-tubulin
β2M
ns
d
Ctrl
Ctrl+XPO5
N
N+XPO5
Relative cytosolic / nuclear pre-miRNA level
pre-miR-21-5p
pre-miR-100-5p
pre-let-7i-5p
e
Relative miRNA / pre-miRNA level
f
Ctrl+Dicer
N+Dicer
miR-27b-3p
let-7i-5p
miR-30a-5p
g
Ctrl+XPO5+Dicer
N+XPO5+Dicer
miR-21-5p

**Supplementary Figure 5. SARS-CoV-2 N protein represses miRNA biogenesis;** related to Fig. 4. **(a)** Heatmap of miRNA expression in BEAS-2B-Ctrl and BEAS-2B-N cells based on small RNA sequencing. **(b)** Quantification of five miRNAs in BEAS-2B-Ctrl and BEAS-2B-N cells. **(c)** Ratio of cytosolic levels of different RNAs to their nuclear levels in BEAS-2B-Ctrl and BEAS-2B-N cells. **(d)** Ratio of cytosolic pre-miRNA levels to nuclear pre-miRNA levels in BEAS-2B-Ctrl and BEAS-2B-N cells transfected with a plasmid expressing XPO5 or control plasmid. **(e)** Ratio of mature miRNA levels to pre-miRNA levels in BEAS-2B-Ctrl and BEAS-2B-N cells. **(f)** Ratio of mature miRNA levels to pre-miRNA levels in BEAS-2B-Ctrl and BEAS-2B-N cells transfected with a Dicer-expressing or control plasmid. **(g)** miRNA levels in BEAS-2B-Ctrl and BEAS-2B-N cells transfected with XPO5-expressing and Dicer-expressing plasmids or a control plasmid. Data in (b–g) are expressed as mean ± SD of three biological replicates. $^{**}p < 0.01$; $^{*}p < 0.05$; ns, not significant ($p > 0.05$; two-tailed unpaired Student's $t$-test). Ctrl: control plasmid; N: N protein. Source data and exact $p$ values are provided in the Source Data file.

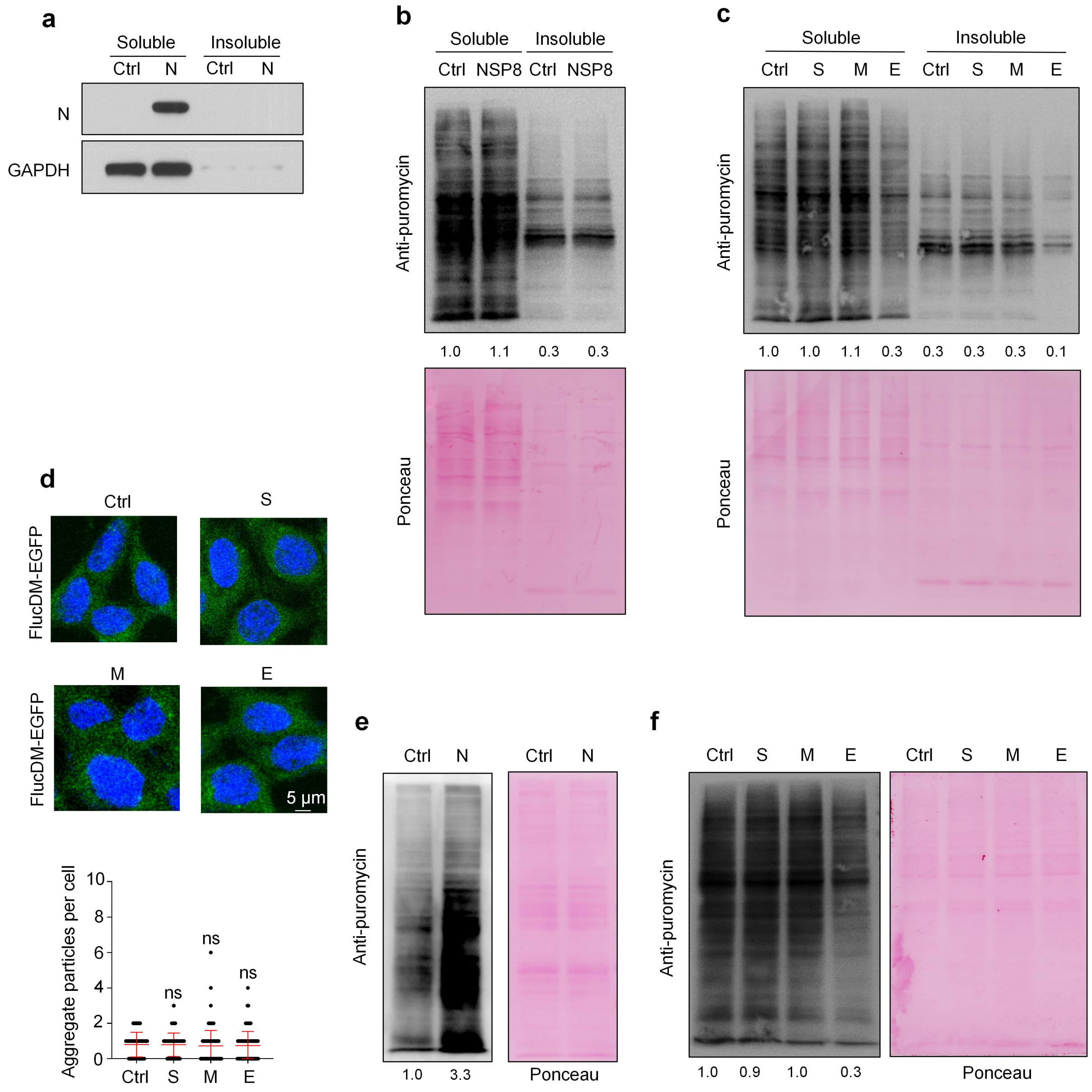


**Supplementary Figure 6. Effects of different proteins encoded by SARS-CoV-2 on**

**proteotoxic stress and protein translation;** related to Fig. 6. **(a)** Distribution of N protein and GAPDH in the soluble and insoluble protein fractions of A549-Ctrl and A549-N cells was determined using immunoblotting. **(b)** Immunoblotting of nascent polypeptides labeled with puromycin in the soluble and insoluble protein fractions of A549 cells transfected with plasmid expressing viral protein NSP8 or a control plasmid. **(c)** Immunoblotting of nascent polypeptides labeled with puromycin in the soluble and insoluble protein fractions of A549 cells transfected with plasmids expressing spike (S), membrane (M), and envelope (E) proteins. **(d)** Proteotoxic stress sensor reporter FlucDM-EGFP were co-transfected with plasmids expressing spike (S), membrane (M), or envelope (E) proteins into A549 cells, and immunofluorescence was detected using confocal microscopy. Data are expressed as mean ± SD. n = 200 cells from three biological repeats. ns, not significant (two-tailed unpaired Student's *t*-test). **(e)** Immunoblotting of nascent polypeptides labeled with puromycin in BEAS-2B-Ctrl and BEAS-2B-N cells. **(f)** Immunoblotting of nascent polypeptides labeled with puromycin of A549 cells transfected with plasmids expressing S, M, E proteins. Ponceau S staining image represents the loading control in (b, c, e, f). The numbers in (b, c, e, f) below the blots represent the relative densitometric quantification of the bands normalized to the corresponding Ponceau S staining bands; the mean values of three independent experiments are shown. Ctrl: control plasmid; N: N protein. Source data and exact *p* values are provided in the Source Data file.

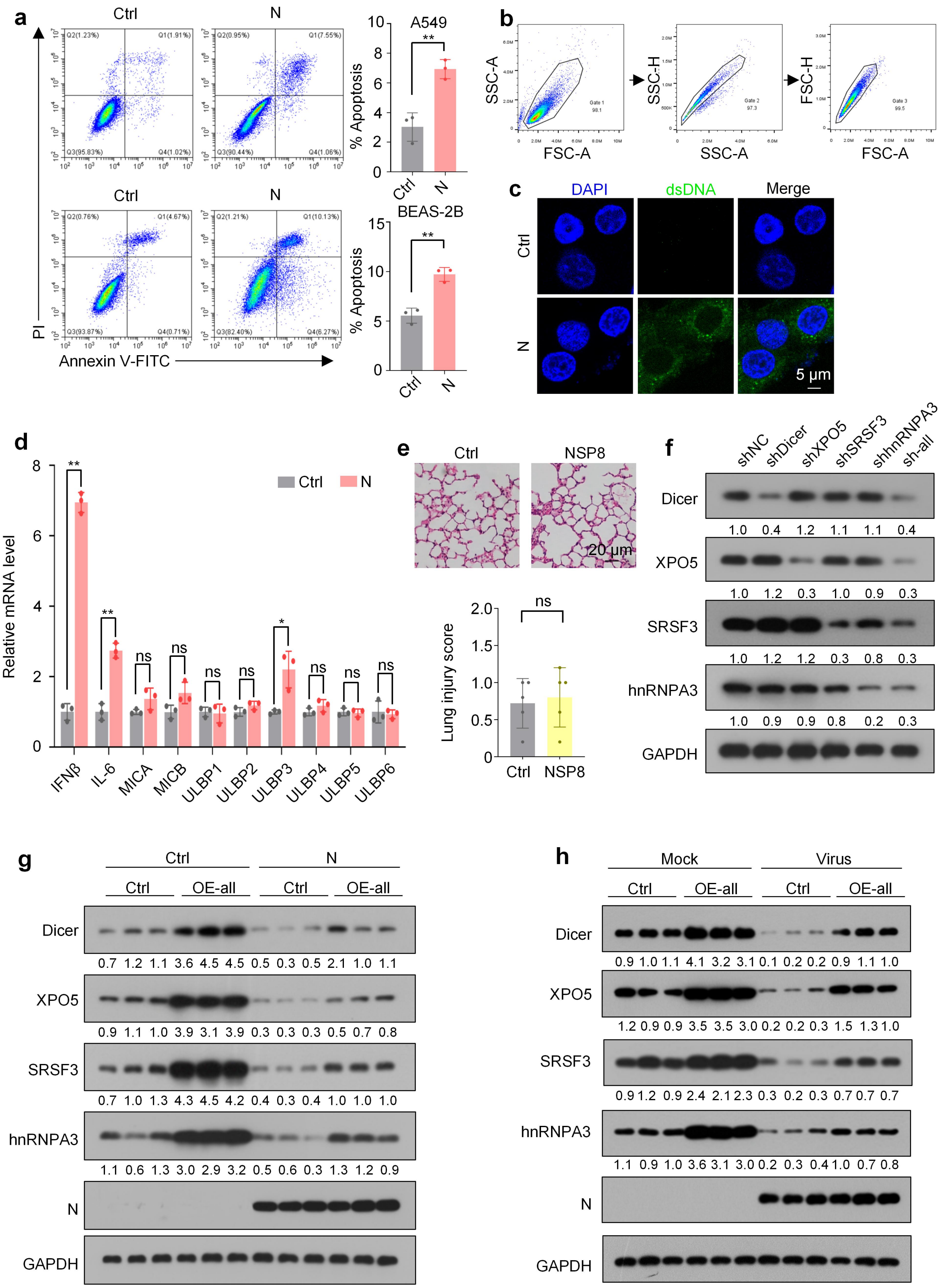
a
Ctrl
N
A549
% Apoptosis
**
Ctrl
N
BEAS-2B
PI
Annexin V-FITC
Q2(1.23%)
Q1(1.91%)
Q3(95.83%)
Q4(1.02%)
Q2(0.95%)
Q1(7.55%)
Q3(90.44%)
Q4(1.06%)
Q2(0.76%)
Q1(4.67%)
Q3(93.87%)
Q4(0.71%)
Q2(1.21%)
Q1(10.13%)
Q3(82.40%)
Q4(6.27%)
b
SSC-A
FSC-A
SSC-H
SSC-A
FSC-H
FSC-A
c
DAPI
dsDNA
Merge
Ctrl
N
5 µm
d
Relative mRNA level
Ctrl
N
**
*
ns
IFNβ
IL-6
MICA
MICB
ULBP1
ULBP2
ULBP3
ULBP4
ULBP5
ULBP6
e
Ctrl
NSP8
20 µm
Lung injury score
ns
Ctrl
NSP8
f
shNC
shDicer
shXPO5
shSRSF3
shhnRNPA3
sh-all
Dicer
1.0 0.4 1.2 1.1 1.1 0.4
XPO5
1.0 1.2 0.3 1.0 0.9 0.3
SRSF3
1.0 1.2 1.2 0.3 0.8 0.3
hnRNPA3
1.0 0.9 0.9 0.8 0.2 0.3
GAPDH
g
Ctrl
N
Ctrl
OE-all
Ctrl
OE-all
Dicer
0.7 1.2 1.1 3.6 4.5 4.5 0.5 0.3 0.5 2.1 1.0 1.1
XPO5
0.9 1.1 1.0 3.9 3.1 3.9 0.3 0.3 0.3 0.5 0.7 0.8
SRSF3
0.7 1.0 1.3 4.3 4.5 4.2 0.4 0.3 0.4 1.0 1.0 1.0
hnRNPA3
1.1 0.6 1.3 3.0 2.9 3.2 0.5 0.6 0.3 1.3 1.2 0.9
N
GAPDH
h
Mock
Virus
Ctrl
OE-all
Ctrl
OE-all
Dicer
0.9 1.0 1.1 4.1 3.2 3.1 0.1 0.2 0.2 0.9 1.1 1.0
XPO5
1.2 0.9 0.9 3.5 3.5 3.0 0.2 0.2 0.3 1.5 1.3 1.0
SRSF3
0.9 1.2 0.9 2.4 2.1 2.3 0.3 0.2 0.3 0.7 0.7 0.7
hnRNPA3
1.1 0.9 1.0 3.6 3.1 3.0 0.2 0.3 0.4 1.0 0.7 0.8
N
GAPDH

**Supplementary Figure 7. SARS-CoV-2 N protein induces pneumonia in mice;** related to Fig. 7. **(a)** Apoptosis of A549-Ctrl and A549-N cells or BEAS-2B-Ctrl and BEAS-2B-N cells measured using Annexin V-FITC and propidium iodide (PI) staining. **(b)** Example of the gating strategy for flow cytometry measurements in (a). FSC-A/SSC-A gate was set to exclude cell debris, and SSC-H/SSC-A and FSC-H/FSC-A gate was then set to exclude doublets. **(c)** Representative confocal microscopy image of cytosolic DNA in A549-Ctrl or A549-N cells (n = 3 biological replicates). **(d)** mRNA levels of *IFNβ, IL-6,* and *NKG2D* ligands in A549-Ctrl or A549-N cells. **(e)** Eight-week-old male mice were intranasally instilled with a plasmid expressing NSP8 protein or a control plasmid, and lung tissues were subjected to HE staining to assess lung injury. **(f)** Immunoblotting of the indicated proteins in the lung tissues from mice that were intranasally instilled with plasmids expressing shNC, shDicer, shXPO5, shSRSF3, and shhnRNPA3 alone or in combination. **(g)** Immunoblotting of the indicated proteins in the lung tissues of mice intranasally instilled with N protein-expressing plasmid or co-instilled with N protein-expressing plasmid and plasmids expressing Dicer, XPO5, SRSF3, and hnRNPA3. **(h)** Eight-week-old K18-hACE2 mice intranasally instilled with plasmids expressing Dicer, XPO5, SRSF3, and hnRNPA3 were intranasally instilled with SARS-CoV-2 or mock-instilled, and their lung tissues were subjected to immunoblotting with the indicated antibodies**.** The numbers below the blots in (f–h) represent the relative densitometric quantification of the bands normalized to corresponding GAPDH bands; the mean values of three independent experiments are shown (f). Representative immunoblots of lung tissues from three out of five mice per group are shown (g, h). Data in (a, d) are expressed as mean ± SD of three biological replicates. Data in (d) are expressed as mean ± SD of five mice. $^{**}p < 0.01$; $^{*}p < 0.05$; ns, not significant ($p > 0.05$; two-tailed unpaired Student's *t*-test). Ctrl: control plasmid; N: N protein; shNC: negative control

shRNA; sh-all: mice instilled with shDicer, shXPO5, shSRSF3, and shhnRNPA3 together; OE-all: mice instilled with Dicer-, XPO5-, SRSF3-, and hnRNPA3-expressing plasmids together. Source data and exact *p* values are provided in the Source Data file.

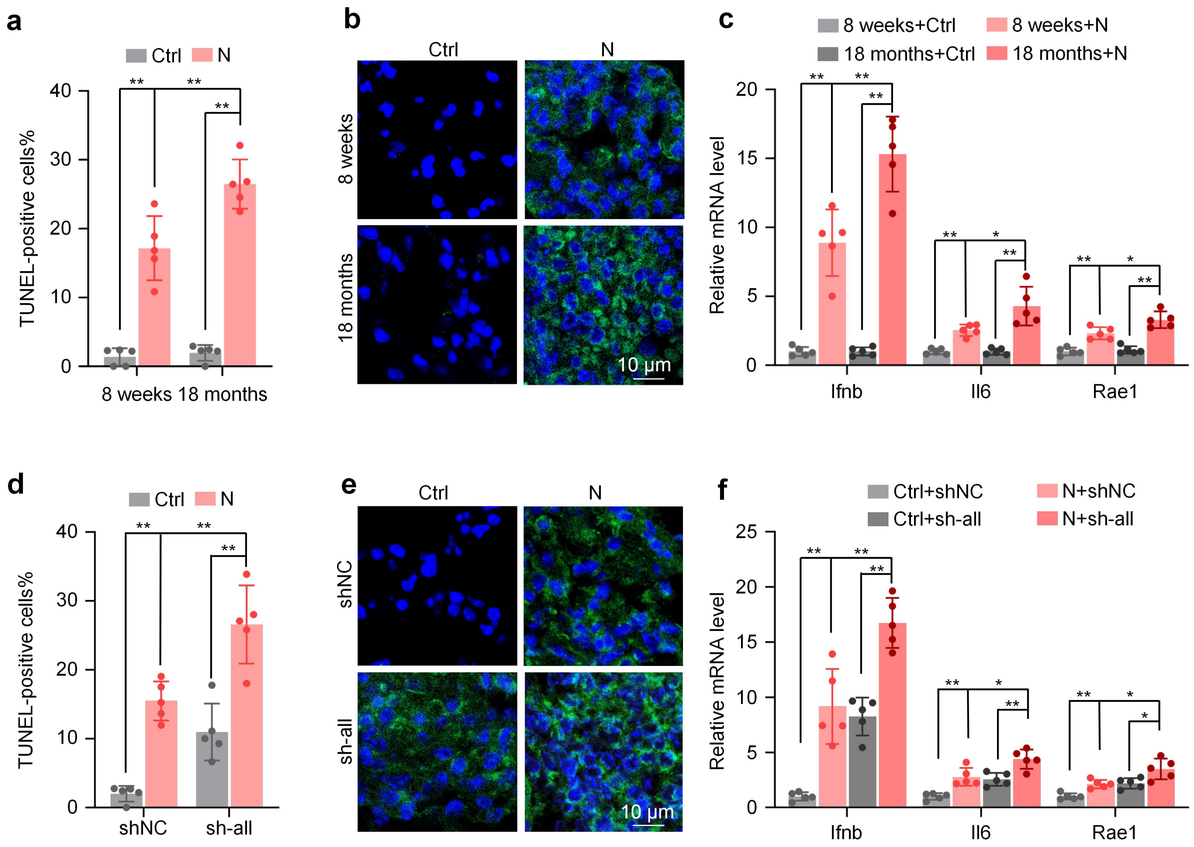


**Supplementary Figure 8. Age-related downregulation of Dicer, XPO5, SRSF3, and hnRNPA3 is associated with the severity of N protein-induced pneumonia;** related to Fig. 8. **(a–c)** Eight-week-old and 18-month-old mice were intranasally instilled with a control or N protein-expressing plasmid, and lung tissues were subjected to terminal deoxynucleotidyl transferase dUTP nick end labeling (TUNEL) assay (a), immunofluorescence with an anti-dsDNA antibody (b), and RT-qPCR analysis of *Ifnb*, *Il6*, and *Rae1* (c). **(d–f)** Lung tissues of 8-week-old mice intranasally instilled with N protein-expressing plasmid or co-instilled with N protein-expressing plasmid and plasmids expressing shRNAs against Dicer, XPO5, SRSF3, and

hnRNPA3 were subjected to TUNEL staining (d), immunofluorescence with anti-dsDNA antibody (e), and RT-qPCR analysis of *Ifnb*, *Il6*, and *Rae1* (f). Data in (a, c, d, f) are expressed as mean ± SD of five mice. ** $p < 0.01$; * $p < 0.05$ (two-tailed unpaired Student's *t*-test). Ctrl: control plasmid; N: N protein; shNC: negative control shRNA; sh-all: mice instilled with shDicer, shXPO5, shSRSF3, and shhnRNPA3 together. Source data and exact *p* values are provided in the Source Data file.

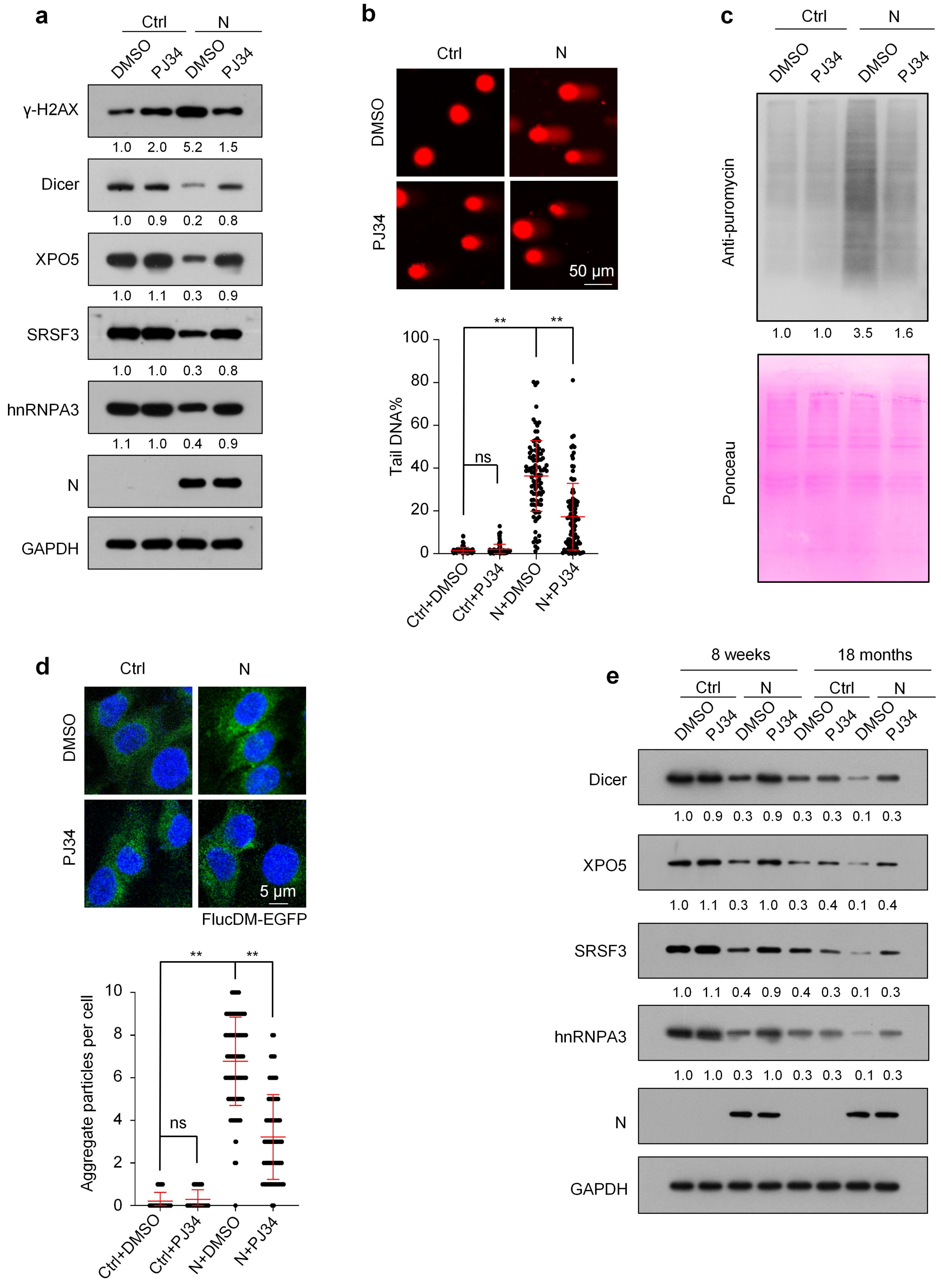


**Supplementary Figure 9. PJ34 relieves N protein-induced pneumonia;** related to Fig. 9. **(a)**

Immunoblotting of the indicated proteins in BEAS-2B-Ctrl or BEAS-2B-N cells treated with or without PJ34 (50 μM) for 2 h. **(b)** Comet assay for detecting DNA damage in A549-Ctrl or A549-N cells treated with or without PJ34 (50 μM) for 2 h. **(c)** Immunoblotting analysis of nascent polypeptides labeled with puromycin in A549-Ctrl or A549-N cells treated with or without PJ34 (50 μM) for 2 h. Ponceau S staining images serve as the loading control. **(d)** Fluorescence image of A549 cells stably expressing the proteotoxic stress sensor reporter FluDM-EGFP transfected with control or N protein-expressing plasmid in the presence or absence of PJ34 (50 μM). **(e)** Mice (8-week-old and 18-month-old) were intranasally instilled with control plasmid or N protein-expressing plasmid and treated with or without PJ34 (10 mg/kg), and lung tissues were subjected to immunoblotting. The numbers below the blots in (a, c, e) represent the relative densitometric quantification of the bands normalized to corresponding GAPDH bands (a, e) or Ponceau S staining bands (c); the mean values of three independent experiments are shown (e). The mean values of three independent experiments are shown in (a, c). Data in (b, d) are expressed as mean ± SD. n = 100 (b) or 200 (d) cells from three biological replicates. $^{**}p < 0.01$; ns, not significant ($p > 0.05$; two-tailed unpaired Student's *t*-test). Ctrl: control plasmid; N: N protein. Source data and exact *p* values are provided in the Source Data file.

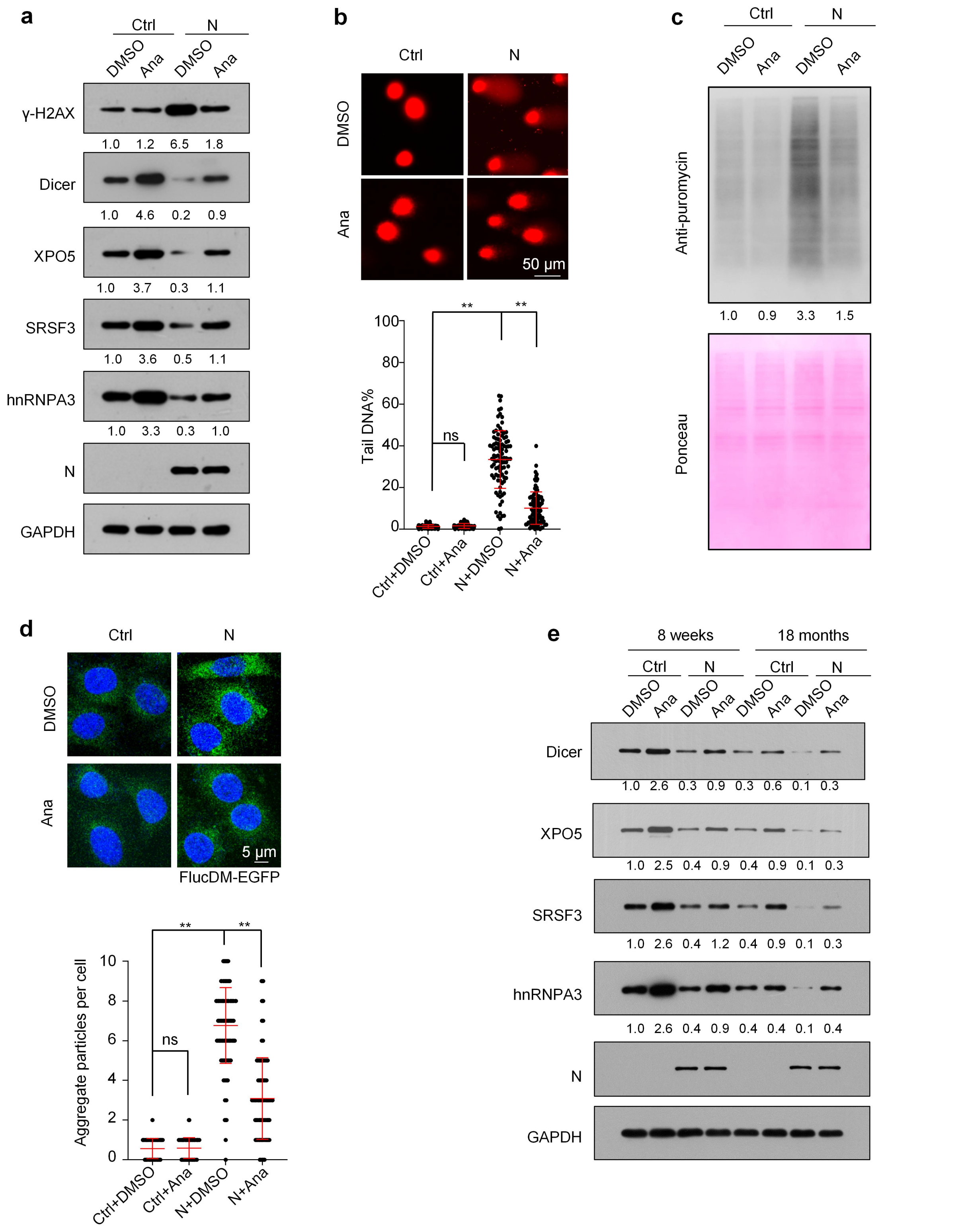


**Supplementary Figure 10. Anastrozole relieves N protein-induced pneumonia;** related to Fig. 10. **(a)** Immunoblotting of the indicated proteins in BEAS-2B-Ctrl or BEAS-2B-N cells treated

with or without anastrozole (5 μM) for 24 h. **(b)** DNA damage was assessed using comet assay in A549-Ctrl or A549-N cells treated with or without anastrozole (5 μM) for 24 h. **(c)** Immunoblotting analysis of nascent polypeptides labeled with puromycin in A549-Ctrl or A549-N cells treated with or without anastrozole (5 μM) for 24 h. Ponceau S staining images serve as the loading control. **(d)** Fluorescence image of A549 cells stably expressing the proteotoxic stress sensor reporter FluDM-EGFP transfected with control or N protein-expressing plasmid in the presence or absence of anastrozole (5 μM). **(e)** Lung tissues of mice (8-week-old and 18-month-old) intranasally instilled with control plasmid or N protein-expressing plasmid and treated with or without anastrozole (20 mg/kg) were subjected to immunoblotting. The numbers below the blots in (a, c, e) represent the relative densitometric quantification of bands normalized to corresponding GAPDH bands (a, e) or Ponceau S staining bands (c); the mean values of three independent experiments are shown (e). The mean values of three independent experiments are shown in (a, c). Data in (b, d) are expressed as mean ± SD. n = 100 (b) or 200 (d) cells from three biological replicates. $^{**}p < 0.01$; ns, not significant ($p > 0.05$; two-tailed unpaired Student's *t*-test). Ctrl: control plasmid; N: N protein; Ana: anastrozole. Source data and exact *p* values are provided in the Source Data file.

## Supplementary Tables

### Supplementary Table 1. Reagents used in this study.

| Name | Vendor | Catalog number |
|---|---|---|
| Roswell Park Memorial Institute 1640 medium | Hyclone | SH30027.01 |
| Fetal bovine serum | Hyclone | SH30084.03 |
| Dulbecco’s modified Eagle medium | Hyclone | SH30022.01 |
| Chloroquine | MedChemExpress | HY-17589A |
| MG132 | MedChemExpress | HY-13259 |
| PJ34 | MedChemExpress | HY-13688A |
| Anastrozole | MedChemExpress | HY-14274 |
| Entranster$^{TM}$-in vivo | Engreen | 18668-11-2 |
| Antigen retrieval solution containing EDTA (pH 9.0) | MXB Biotechnologies | MVS-0098 |
| 3% $H_2O_2$ | ZSGB-BIO | ZLI-9311 |
| Protein blocking solution | ZSGB-BIO | ZLI-9021 |
| 4′,6-diamidino-2-phenylindole | Sigma-Aldrich | D9542 |
| Lipofectamine 2000 | Thermo Fisher Scientific | 11668016 |
| Polybrene | Sigma-Aldrich | TR-1003 |
| Puromycin | MedChemExpress | HY-B1743A |
| Ponceau S staining solution | Thermo Fisher Scientific | A40000279 |
| Low-melting-point agarose | Solarbio | A8350 |
| Agarose | Solarbio | A8201 |
| Ethidium bromide | Sigma-Aldrich | E7637 |
| Protease inhibitor cocktail tablets | Roche | COEDTAF-RO |
| Phosphatase inhibitor cocktail | APExBIO Technology LLC | K1015 |
| Clarity western enhanced chemiluminescence substrate | Bio-Rad Laboratories | 1705061 |
| TRIzol reagent | Life Technologies | 15596026 |
| Protein A/G magnetic beads | Selleck Chemicals | B23202 |
| RNase A | TaKaRa | 2158 |
| RNase T1 | Thermo Fisher Scientific | AM2283 |
| RNase V1 | Life Technologies | AM2275 |
| RNase I | Life Technologies | EN0601 |
| RNase inhibitor | Vazyme Biotech Co., Ltd | R301 |
| Proteinase K | Sigma-Aldrich | P2308 |

**Supplementary Table 2. Plasmids used in this study.**

| Name | Vendor | Catalog number |
|---|---|---|
| pLVX-EF1alpha-SARS-CoV-2-N-2xStrep-IRES-Puro | Addgene | 141391 |
| pLVX-EF1alpha-SARS-CoV-2-E-2xStrep-IRES-Puro | Addgene | 141385 |
| pLVX-EF1alpha-SARS-CoV-2-M-2xStrep-IRES-Puro | Addgene | 141386 |
| pLVX-EF1alpha-SARS-CoV-2-S-2xStrep-IRES-Puro | HITRO BioTech | Custom-made |
| pLVX-EF1alpha-SARS-CoV-2-NSP8-2xStrep-IRES-Puro | HITRO BioTech | Custom-made |
| pLVX-EF1alpha-2xStrep-IRES-Puro | HITRO BioTech | Custom-made |
| pLVX-3×FLAG-hACE2 | HITRO BioTech | Custom-made |
| CMV-LUC2CP/ARE | Addgene | 62857 |
| CMV-LUC2CP/intron/ARE | Addgene | 62858 |
| pCI-neo Fluc-EGFP | Addgene | 90170 |
| pCI-neo FlucDM-EGFP | Addgene | 90172 |
| pMD2.G | Addgene | 12259 |
| psPAX2 | Addgene | 12260 |
| pDESTmycDICER | Addgene | 19873 |
| pKmyc-Exp5 | Addgene | 12552 |
| pCDH-CMV-MCS-EF1-Puro-SRSF3(h) | Wuhan GeneCreate | Custom-made |
| pCDH-CMV-MCS-EF1-Puro-hnRNPA3(h) | Wuhan GeneCreate | Custom-made |
| pCDH-CMV-MCS-EF1-Puro-RNase H1(h) | Wuhan GeneCreate | Custom-made |
| pCDH-CMV-MCS-EF1-Puro-Dicer(m) | Wuhan GeneCreate | Custom-made |
| pCDH-CMV-MCS-EF1-Puro-XPO5(m) | Wuhan GeneCreate | Custom-made |
| pCDH-CMV-MCS-EF1-Puro-SRSF3(m) | Wuhan GeneCreate | Custom-made |
| pCDH-CMV-MCS-EF1-Puro-hnRNPA3(m) | Wuhan GeneCreate | Custom-made |
| pGL3-Control Vector | Promega | E1741 |
| pRL-CMV Vector | Promega | E2261 |
| pLKO.1-shNC-puro | Tsingke Biotech | Custom-made |
| pLKO.1-shFluc-puro | Tsingke Biotech | Custom-made |
| pLKO.1-shDicer(m)-puro | Tsingke Biotech | Custom-made |
| pLKO.1-shXPO5(m)-puro | Tsingke Biotech | Custom-made |
| pLKO.1-shSRSF3(m)-puro | Tsingke Biotech | Custom-made |
| pLKO.1-shhnRNPA3(m)-puro | Tsingke Biotech | Custom-made |

**Supplementary Table 3. Sequences (5′–3′) of siRNAs and primers used in this study.**

| Gene | | siRNA and shRNA targeting sequences |
|---|---|---|
| shNC/siNC | | TTCTCCGAACGTGTCACGT |
| shFluc | | CTTACGCTGAGTACTTCGA |
| Human sip62-1 | | GCATTGAAGTTGATATCGAT |
| Human sip62-2 | | GGACCCATCTGTCTTCAAATT |
| Human siDicer-1 | | AAGAGTTTACTAAGCACCAGG |
| Human siDicer-2 | | AAGGCTTACCTTCTCCAGGCT |
| Human siXPO5-1 | | GATGCTCTGTCTCGAATTGTA |
| Human siXPO5-2 | | CCAGATGTTTCGAACACTAAA |
| Human siSRSF3-1 | | AGAGCTAGATGGAAGAACATT |
| Human siSRSF3-2 | | GCAACAAGACGGAATTGGATT |
| Human sihnRNPA3-1 | | TCTTTACTTGTTAACTCACAA |
| Human sihnRNPA3-2 | | GGAGGGAACTTTGGAGGTGTT |
| Mouse shDicer | | AGATCACCGTCTCTAGAAA |
| Mouse shXPO5 | | GATTTGATTTCGATAGTGATT |
| Mouse shSRSF3 | | GGAAATAATGGAAACAAGAA |
| Mouse shhnRNPA3 | | TTAAAGAGGATACGGAAGA |
| **Gene** | **Forward/Reverse** | **Primer sequence** |
| Human *IFNB* | Forward | TCTCCTCAGGGATGTCAAAG |
| | Reverse | CAACAAGTGTCTCCTCCAAAT |
| Mouse *Ifnb* | Forward | TCCTGCTGTGCTTCTCCACCACA |
| | Reverse | AAGTCCGCCCTGTAGGTGAGGTT |
| Human *IL6* | Forward | CAATCTGGATTCAATGAGGAGAC |
| | Reverse | CTCTGGCTTGTTCCTCACTACTC |
| Mouse *Il6* | Forward | GAGACTTCACAGAGGATACCAC |
| | Reverse | CAGTGCATCATCGCTGTTCATAC |
| Human *MICA* | Forward | CTTGGCCATGAACGTCAGG |
| | Reverse | CCTCTGAGGCCTCRCTGCG |
| Human *MICB* | Forward | ACCTTGGCTATGAACGTCACA |
| | Reverse | CCCTCTGAGACCTCGCTGCA |
| Human *ULBP1* | Forward | GTACTGGGAACAAATGCTGGAT |
| | Reverse | AACTCTCCTCATCTGCCAGCT |
| Human *ULBP2* | Forward | TTACTTCTCAATGGGAGACTGT |
| | Reverse | TGTGCCTGAGGACATGGCGA |
| Human *ULBP3* | Forward | CCTGATGCACAGGAAGAAGAG |

| | | |
|---|---|---|
| | Reverse | TATGGCTTTGGGTTGAGCTAAG |
| Human *ULBP4* | Forward | CGCCTTCTTTTGTTTCTGCTG |
| | Reverse | CCTGAGGTCTCGCCCCACT |
| Human *ULBP5* | Forward | CCTGGAAAGCACAGAACCCA |
| | Reverse | ACTGAGCTGCCAAGATCCAC |
| Human *ULBP6* | Forward | TCATCCCTAAGTTCAGACCTGG |
| | Reverse | GGACTGACGGGTGTGACTG |
| Mouse *H60* | Forward | GATGAACAGCATAGCATCTACT |
| | Reverse | CCTCATATCTTTCTCTAGGTTCT |
| Mouse *Rae1* | Forward | CAGTGACCAAGCGCCATCAT |
| | Reverse | ACCTAAGAGAGTGTGCATCATC |
| Human *GAPDH* | Forward | CTGGCGTCTTCACCACCATGG |
| | Reverse | CATCACGCCACAGTTTCCCGG |
| Human *U6* | Forward | AACGCTTCACGAATTTGCGT |
| | Reverse | CTCGCTTCGGCAGCACA |
| Human *18S* | Forward | ATGCAAACAGAGTCCCGACCAGA |
| | Reverse | GCGCAGAACCTACCATCGACAG |
| Human *β-actin* | Forward | AAGATGACCCAGATCATGTTTGAG |
| | Reverse | GCAGCTCGTAGCTCTTCTCCAG |
| Human *β-tubulin* | Forward | TGGACTCTGTTCGCTCAGGT |
| | Reverse | TGCCTCCTTCCGTACCACAT |
| Human *β2M* | Forward | GAGGCTATCCAGCGTACTCCA |
| | Reverse | CGGCAGGCATACTCATCTTTT |
| Mouse *Gapdh* | Forward | AACTTTGGCATTGTGGAAGG |
| | Reverse | CACATTGGGGGTAGGAACAC |
| Human *Dicer* | Forward | TCCACGAGTCACAATCAACACGG |
| | Reverse | GGGTTCTGCATTTAGGAGCTAGATGAG |
| Mouse *Dicer* | Forward | GCCAAGAAAATACCAGGTTGAGC |
| | Reverse | GCGATGAACGTCTTCCCTGAG |
| Human *XPO5* | Forward | ACGACGGGTGCATGGCTTCC |
| | Reverse | TTCGGCGCTTGTCAGCCACT |
| Mouse *Xpo5* | Forward | ACAAATTGCCATCGTCAGACA |
| | Reverse | CTCCAATCGGGACATGCTGT |
| Human *hnRNPA3* | Forward | GAAGGAGCTCTTCGCCTTTT |
| | Reverse | CAAACCTTACCCAGCCAGAA |
| Mouse *Hnrnpa3* | Forward | GAGGGCCATGATCCAAAGGAA |

| | | |
|---|---|---|
| | Reverse | CACAAGAGTAGGTCACAAAACCA |
| Human *SRSF3* | Forward | AGCTGATGCAGTCCGAGAG |
| | Reverse | GGTGGGCCACGATTTCTAC |
| Mouse *Srsf3* | Forward | GACCACTCAGAAGTGTGTGGG |
| | Reverse | TCCTCAAATTCGACGAAAGCAAA |
| Human *ARGLU1* | Forward | AGCTGCTGATGCAGGTATTG |
| | Reverse | GTCCAGTGTCCTGCAGAGTG |
| Human *ARGLU1-IR* | Forward | GAAGAACTCGAGCGACAGAGA |
| | Reverse | CTGTTCTTCGGCCAGTTTG |
| Human *CCN1* | Forward | AAGATGCTTGTGGTTTGGCCC |
| | Reverse | TTCCGTATGCGCTTTCGTTG |
| Human *CCN1-IR* | Forward | CAAGGGGCTGGAATGCAACT |
| | Reverse | TTCTCGTCAACTCCACCTCG |
| Human *TERT* | Forward | ACCTTCCTCAGGACCCTGGT |
| | Reverse | ATCTGAACAAAAGCCGTGCC |
| Human *TERT-IR* | Forward | GCCAATCCCAAAGGGTCAGA |
| | Reverse | TCGGGTTCAGAGGGACTCAT |

**Supplementary Table 4. Antibodies used in this study.**

| **Antibody** | **Vendor** | **Catalog number** |
|---|---|---|
| Rat anti-Strep-tag II | Abcam | ab252885 |
| Rabbit anti-SARS-CoV-2 (2019-nCoV) Nucleocapsid | Sino Biological Inc. | 40588-RC02 |
| Rabbit anti-ACE2 | Proteintech | 21115-1-AP; RRID:AB_10732845 |
| Rabbit anti-SRSF3 | Abcam | ab198291 |
| Mouse anti-Dicer | Abcam | ab14601; RRID:AB_443067 |
| Rabbit anti-Exportin 5 | Cell Signaling Technology | 12565; RRID:AB_2737081 |
| Rabbit anti-RNase H1 | Proteintech | 15606-1-AP; RRID:AB_2238624 |
| Rabbit anti-hnRNPA3 | Proteintech | 25142-1-AP; RRID:AB_2879921 |
| Rabbit anti-Phospho-ATM (Ser1981) | Cell Signaling Technology | 13050; RRID:AB_2798100 |
| Rabbit anti-ATM | Proteintech | 27156-1-AP; RRID:AB_2880780 |
| Rabbit anti-Phospho-ATR (Ser428) | Cell Signaling Technology | 2853; RRID:AB 2290281 |
| Rabbit anti-ATR | Proteintech | 19787-1-AP; RRID:AB_10639516 |
| Rabbit anti-Phospho-Chk1 (Ser345) | Cell Signaling Technology | 2348; RRID:AB_331212 |

| | | |
|---|---|---|
| Rabbit anti-Chk1 | Proteintech | 25887-1-AP; RRID:AB_2880283 |
| Rabbit anti-Phospho-Chk2 (Thr68) | Cell Signaling Technology | 2661; RRID:AB_331479 |
| Rabbit anti-Chk2 | Cell Signaling Technology | 2662; RRID:AB_2080793 |
| Rabbit anti-Phospho-Histone H2A.X (Ser139) | Cell Signaling Technology | 2577; RRID:AB_2118010 |
| Rabbit anti-Histone H2A.X Polyclonal | Proteintech | 10856-1-AP; RRID:AB_2114985 |
| Mouse anti-SQSTM1/p62 | Cell Signaling Technology | 88588; RRID:AB_2800125 |
| Rabbit anti-SQSTM1/p62 | Proteintech | 18420-1-AP; RRID:AB_10694431 |
| Rabbit anti-LC3 | Proteintech | 14600-1-AP; RRID:AB_2137737 |
| Rabbit anti-Phospho-SAPK/JNK (Thr183/Tyr185) | Cell Signaling Technology | 9251; RRID:AB_331659 |
| Rabbit anti-JNK1/2/3 | Proteintech | 28007-1-AP; RRID:AB_2881035 |
| Rabbit anti-Phospho-p70(S6K) (Thr389) | Proteintech | 28735-1-AP; RRID:AB_2918197 |
| Mouse anti-p70(S6K) | Proteintech | 66638-1-Ig; RRID:AB_2881997 |
| Rabbit anti-Phospho-4EBP1 (Ser65/Thr70) | Abmart | TA2308 |
| Mouse anti-4EBP1 | Proteintech | 60246-1-Ig; RRID:AB_2881368 |
| Rabbit anti-CD3 | Proteintech | 17617-1-AP; RRID:AB_1939430 |
| Rabbit anti-CD22 | Proteintech | 21894-1-AP; RRID:AB_2878937 |
| Rabbit anti-CD68 | Proteintech | 28058-1-AP; RRID:AB_2881049 |
| Rabbit anti-Ly6G | Abcam | ab238132; RRID: AB_2923218 |
| Mouse anti-GAPDH | Proteintech | 60004-1-Ig; RRID:AB_2107436 |
| Mouse anti-DNA antibody, double-stranded, clone AE-2 | Sigma-Aldrich | MAB1293; RRID:AB_11215105 |
| Mouse anti-puromycin | Sigma-Aldrich | MABE343; RRID:AB_2566826 |
| Mouse anti-DNA-RNA hybrid (R-loop) antibody, clone S9.6 | Sigma-Aldrich | MABE1095; RRID:AB_2861387 |
| Rabbit IgG | Beyotime Biotechnology | A7058 |
| Mouse IgG | Beyotime Biotechnology | A7050 |
| Mouse anti-rabbit IgG (Conformation Specific) (L27A9) mAb (HRP Conjugate) | Cell Signaling Technology | 5127; RRID:AB_10892860 |
| Goat anti-rabbit IgG (H+L) secondary antibody, HRP conjugate | Biosharp | BL003A; RRID:AB_2827666 |
| Goat anti-mouse IgG (H+L) secondary antibody, HRP conjugate | Biosharp | BL001A; RRID:AB_2827665 |
| Goat anti-Rabbit IgG (H+L) Cross-Adsorbed Secondary Antibody, Alexa Fluor™ 488 | Thermo Fisher Scientific | A-11008; RRID:AB_143165 |

| | | |
|---|---|---|
| Rabbit anti-Rat IgG (H+L) secondary antibody, HRP conjugate | Boster | BA1058; RRID:AB_10891896 |
| Goat anti-Rabbit IgG (H+L) Cross-Adsorbed Secondary Antibody, Alexa Fluor™ 555 | Thermo Fisher Scientific | A-21428; RRID:AB_2535849 |
| Goat anti-Mouse IgG (H+L) Cross-Adsorbed Secondary Antibody, Alexa Fluor™ 488 | Thermo Fisher Scientific | A-11001; RRID:AB_2534069 |
